# Supplementary material for: Structure-from-motion photogrammetry demonstrates variability in coral growth within colonies and across habitats
Source: PLoS One. 2022 Nov 16;17(11):e0277546. doi: 10.1371/journal.pone.0277546 (PMC9668137; doi:10.1371/journal.pone.0277546)
Supplement: S1 Table — (PDF) [file pone.0277546.s001.pdf]

S1 Table: **Workflows for 3D model construction and measurement of coral colony growth rates using Metashape, CloudCompare and Meshmixer**

Table A: Workflow for 3D model construction from overlapping photographs in Agisoft Metashape Professional 1.8.2. This table has been copied from Lange & Perry (2020). Time estimated for each step is based on a user accustomed to the program and method. Time necessary for processing steps (in green) depends on CPU, GPU and RAM of the used work station and values given here are based on using a HP EliteBook (2.1 GHz CPU, 16 GB RAM, 6.5 MB GPU). If several models are loaded into the same workspace as separate chunks, 'Alignment' and 'Dense Cloud' generation can be processed for all those models in one step ('Workflow' – 'Batch process' – 'Add Job' and choose settings).

Steps	Menu	Function	Action	Time (min)
Photo setup, Alignment				
1	Main Menu - Workflow	Add Photos	Navigate to directory with photos. Select and add all colony photos. Ensure consistent orientation and good quality of all photos (landscape).	2
2	Photo Panel - right click on any photo	Estimate Image Quality – All Cameras	Check values (change view to details), disable cameras if <0.3, check cameras if <0.5 and remove if blurry	2
3	Main Menu - Workflow	Align Photos	Settings: high, generic preselection, 40,000, 4,000, do not apply masks	15-20
4	Reference Panel	Optimize Cameras	☑ check all except k4, b1, b2, p3, p4 (default)	1
			Total time	**20-25**
Error reduction				
5	Main Panel	Resize Region	If area around colony is very large, decrease the size of the bounding box	
6	Main Menu - Model Gradual Selection	Reconstruction Uncertainty (Pixel matching Errors)	Set level 10-15 (if more than 30% of pts are selected, increase the level)<br>Delete the points, Optimize cameras, ☑ check all except k4, b1, b2, p3, p4<br>If necessary repeat until level is closer to 10-15 (e.g. 1st step: 15, 2nd step: 12)	1
7	Main Menu - Model Gradual Selection	Projection Accuracy (Pixel matching Errors)	Set level 3-5 (if more than 30% of pts are selected, increase the level to 5-9)<br>Delete the points, Optimize cameras, ☑ check all except k4, b1, b2, p3, p4<br>Repeat 1-2 times until level is closer to 3-5 (e.g. 1st step: 7, 2nd step: 4)<br>Monitor in references, cameras: Projections goal >100, Error (pix) goal = 0.3-0.7	1
8	Reference Panel	Settings - Tighten Tie Point Accuracy	Change tie point accuracy from 1 to 0.1<br>Optimize cameras, ☑ check all<br>Monitor SEUW in console which should get closer to 1	1
9	Main Menu - Model Gradual Selection	Reprojection Error (Pixel Residual Errors)	Set level 0.3-0.5 (if more than 10% of pts are selected, increase the level)<br>Delete the points, Optimize cameras, ☑ check all<br>Repeat 2-3x on same level until reaching it without having to delete points<br>Monitor: Projections goal >100, Error (pix) goal = 0.3-0.7, SEUW (console) = 1.0	2
			Total time	**5**
Dense Point Cloud, Scaling, Export				
10	Workflow	Build Dense Cloud	Settings: medium, aggressive, do not reuse depth maps, point colours	30-35
11	Main Menu - Tools<br><br>References Panel	Markers - Detect markers	Circular 12 bit, tolerance 50. Or add markers manually (right click, add marker).<br>Projections should be >3 for markers used for scaling<br>Mark two markers, 'Create Scale Bar', add distance (in 'View Source' mode), update, check for errors (in 'View errors' mode)	3
12	Main Menu - File	Save as	Save project as .psx	1
13	Main Menu - File	Export - Export Points	Export dense point cloud as .ply	1
			Total time	**35-40**

Table B: Workflow for 3D model comparison and linear growth rate measurements in CloudCompare v2.10.2 (Zephyrus). This table has been updated from Lange & Perry 2020, including the use of the M3C2 plugin for average linear extension measurements and the creation of surface meshes using the Poisson reconstruction plugin. Time estimated for each steps is based on a user accustomed to the program and method and can be longer, especially if alignment of models is difficult. Abbreviations: avg. – average, SD – standard deviation.

Steps	Menu	Function	Action	Time (min)
Align point clouds, measure growth rates				
1	Main Panel	Open	Navigate to directory with point clouds. Select and add point clouds (.ply) of the same colony of two subsequent years (=A older, B more recent cloud).	1
2	DB Tree Panel<br>Main Panel	Align Point clouds	Highlight 1 cloud, translate/rotate, move cloud next to other cloud in similar orientation	5
			Highlight both clouds (ctrl+left click), align the two clouds by picking at least 3 equivalent point pairs (not located in one line or plane), align, ok	
			If the fit is not perfect, adjust alignment by translate/rotate 1 cloud	
			Highlight one cloud, segment around the outline of the cloud by polygonal selection, segment in/out, ok, switch off cloud.remaining. Repeat for other cloud.	
3	DB Tree Panel<br>Main Panel	Compute M3C2 distance	Highlight both clouds.segmented, compute M3C2 distance, set A as Cloud #1 and B as Cloud #2, for core points use cloud #1. Click 'Guess parameters' to update projection parameters but make sure max depth includes maximum distance between clouds	1
			Highlight M3C2 output scale, filter points by values and export 0 to max. Highlight new subset, Distribution: Gauss displays average distance (± SD) = **average linear extension**	1
4	DB Tree Panel<br>Properties Panel<br>Main Panel	Measure point distances	Highlight both clouds.segmented, compute cloud/cloud distance, choose cloud A as reference, compute, ok	3-5
			Highlight cloud B, display Scalar Field, potentially change colour scale (move sliders in Properties Panel) to clearly display areas of maximum extension	
			use to measure point distances in regions of maximum extension (upwards facing surface/ bumps/ridges/columns/branch tips etc.), click after each point to save labels (n≥15), calculate average (± SD) = **maximum linear extension**	
5	DB Tree Panel<br>Main Panel	Calculate 2.5D Volume	Highlight both clouds, translate/rotate until the top view displays the perspective of main growth direction	1
			Go to Tools > Volume > Compute 2.5D Volume. Choose cloud A as 'Before' and cloud B as 'After', Empty cells: leave empty, Grid step: 0.001, projection dir.: Z, cell height: maximum height. Click update. From results table copy added volume = **vertical volume increase**	
6	DB Tree Panel<br>Main Panel	Create mesh and measure surface area	Highlight 1 cloud, go to Plugins, PoissonRecon: Octree depth 11, output density as SF, ok. This will produce a closed mesh or add surface around the colony perimeter.	3-5
			Move slider in 'SF display params' until selection only includes the colony surface, filter points by values and export the selection as new mesh	
			Go to Edit > Mesh > Measure surface, the results are displayed in the console, copy and from area of both clouds calculate = **surface area increase**	
7	Main Panel	Export meshes	Highlight segmented mesh, save as .ply, ASCII format. repeat for other mesh.	1
			Total time	**15-20**

Table C: Workflow for direct volume increase measurements in Meshmixer v3.5.474 (Audodesk). Time estimated for each steps is based on a user accustomed to the program and method and can be longer, especially if joining of model boundaries is difficult. Abbreviations: avg. – average, SD – standard deviation.

Steps	Menu	Function	Action	Time (min)
Measure volume changes				
1	Main Panel	Import	Navigate to directory with meshes. Select and add meshes (.ply, ASCII format) of the same colony of two subsequent years (=A older, B more recent mesh).	1
2	Object browser and Main Panel	Smooth boundaries	Highlight 1 mesh in the Object Browser (you can hide the other by clicking on the eye symbol), Analysis > Inspector will light up holes and disconnected components of the mesh. Left click on pink, blue and red balls to repair small holes and disconnected mesh parts, but do not repair the boundary of the mesh. Big holes in the mesh can be filled using the bridge tool.<br>If red/pink balloons (i.e. non-manifold/disconnected) are indicated for the boundaries, the problematic part of the boundary (loops, detached parts) will have to be located, highlighted and repaired using Edit > Erase&Fill or Discard.<br>Repeat for other mesh (toggle in Object Browser).	5-15
			Highlight 1 mesh, Select, decrease brush size, double click on blue boundary line to highlight boundary faces in orange (if whole mesh lights up in orange press S and try again). Go to Modify > Optimize boundary. Then Modify > Smooth boundary (Smoothness = 100, Preserve Shape = 0, untick all boxes) > Accept. Repeat for other mesh	
			Highlight mesh A, Select, double click on mesh to highlight whole mesh in orange. Go to Edit > Flip normals to turn mesh inside out	
3	Object browser and Main Panel	Join meshes	Select both meshes in Object browser (Ctrl+left click). Go to Edit > Combine	3-5
			Select, highlight both boundaries by double clicking on both blue lines. Go to Edit > Join. This step can take a few minutes, when completed check if space between meshes is bridged and accept. If the joining is not successful but introduces unwanted artefacts, cancel and try to find the fault along the colony boundaries (remove loops, holes), possibly close gap with the bridge tool.	
4	Main Panel	Measure volume	Analysis, Stability, copy volume (here indicated as mm^3 but depends on import scale – in our case in m^3), convert to cm^3 = **volume increase**	1
			Total time	**10-20**
