# Supplementary material for: Structure-from-motion photogrammetry demonstrates variability in coral growth within colonies and across habitats
Source: PLoS One. 2022 Nov 16;17(11):e0277546. doi: 10.1371/journal.pone.0277546 (PMC9668137; doi:10.1371/journal.pone.0277546)
Supplement: Attachment — Submitted filename: review PLOSONE.odt [file pone.0277546.s003.odt]

The Authors presented a methodological approach for monitoring corals’ morphology changes over time using the structure from motion technology and open-source software to estimate the distance between point clouds of the same colony obtained from two monitoring events.
The corals scanning was carried out by positioning reference dimension items close to the subject. No fixed reference points were used during the monitoring to align point clouds generated from different monitoring events.
The alignment of two-point clouds was done manually and using a tool (S2 table, step 2 of the Sup. Materials). The error associated with this methodological step is not quantified or is not possible to be quantified, but it affects the measured coral growth rates.

The lacking areas to revise are:
- Need of a map to visually see the location where the experiment was carried out.
- Please provide details on how the camera was used for the survey (manual mode or automatic setting? which are the parameters used for the shooting (i.e. Aperture, exposition, zoom))
- Please provide detail on the weather conditions during the surveys. Do you have estimated horizontal visibility at each location?
- Please change the figures and the plot with high-resolution images (i.e., 300dpi)
