# Supplementary material for: Structure-from-motion photogrammetry demonstrates variability in coral growth within colonies and across habitats
Source: PLoS One. 2022 Nov 16;17(11):e0277546. doi: 10.1371/journal.pone.0277546 (PMC9668137; doi:10.1371/journal.pone.0277546)
Supplement: Attachment — Submitted filename: Response to Reviewers.docx [file pone.0277546.s005.docx]

Response to Reviewers:

Editor:

Authors should discuss the limits of their approach and how much these limits may affect their results. Reviewer 1 lamented the lack of fixed reference points. Commenting upon the potential error deriving from the procedure adopted is deemed necessary before acceptance.

Thank you for your comments. In response to Reviewer 1 we have discussed the limitations of our approach and the lack of fixed reference points in more detail in the revised manuscript (also see responses below). We have also made the other adjustments suggested by both Reviewer 1 and Reviewer 2.

Reviewer #1:

The Authors presented a methodological approach for monitoring corals’ morphology changes over time using the structure from motion technology and open-source software to estimate the distance between point clouds of the same colony obtained from two monitoring events.
The corals scanning was carried out by positioning reference dimension items close to the subject. No fixed reference points were used during the monitoring to align point clouds generated from different monitoring events.
The alignment of two-point clouds was done manually and using a tool (S2 table, step 2 of the Sup. Materials). The error associated with this methodological step is not quantified or is not possible to be quantified, but it affect the measured coral growth rates.

Thank you for your comments. We agree that fixed reference points would be preferable to align point clouds of subsequent years. However, in lots of situations (here for example working in a protected area), the deployment of permanent structures is not possible. We therefore use common points in the surrounding of each colony which are visible in the 3D reconstructions to align the two clouds. The error for this procedure has been quantified in our prior study Lange and Perry (2020), *Methods in Ecology and Evolution* and caused less than 1 mm deviation in growth rates.

We apologize for not having described this issue in more detail and now discuss the potential error resulting from manual alignment in both the Method section (123-127) and the Discussion section (L386-392).

The lacking areas to revise are:

- Need of a map to visually see the location where the experiment was carried out.

We have added a map of the study site as Figure 1 and renamed all other Figures accordingly.

- Please provide details on how the camera was used for the survey (manual mode or automatic setting? which are the parameters used for the shooting (i.e. Aperture, exposition, zoom))

We have added this information (automatic underwater settings, no zoo, no flash) to the Method section in L.107.

- Please provide detail on the weather conditions during the surveys. Do you have estimated horizontal visibility at each location?

We have added this information (horizontal visibility >10 m at Catedral, 7-8 m at Tanchacte, with more variable and lower visibility for parts of the year) to the method section in L.96-97.

- Please change the figures and the plot with high-resolution images (i.e., 300dpi)

The Figures are supplied in high resolution which is visible when downloading the Figures with the associated links.

Reviewer #2:

The study has great interest and a high degree of novelty. It is very well thought out and resolved. Congratulations to the authors. I have only commented on some details (attached file) that could be considered in a new version of the manuscript.

Thank you very much for your positive review and your helpful comments. We have added your suggestions to the manuscript and answered to each of your comments below.

Perhaps the lack of knowledge in deep water species (outside the diving range) is higher.

Certainly. But even for shallow water species the lack of growth data is astonishing.

Perhaps parameters such as biodiversity indices or estimation of the proportion of reef-forming species could be added here.

Yes, the assemblage itself is of course important and mentioned in the previous sentence. In these novel assemblages it is then interesting to assess how functions change. To assess the function carbonate production, we need growth rates.

Some references could be added: Bennecke, Olinger, Prado

These are very interesting studies and I have read and cited them in my previous study. However, as I specifically talk about coral colony growth here I would prefer not to cite the studies at this location.

Mention at some point in the manuscript that the possible variation in growth rate within the period of time evaluated has not been considered. This does not have to be uniform and different factors can affect growth within a period of time. In addition, the speed of growth is usually related to the age of the specimens (higher growth ratio in young specimens). Perhaps it is convenient to comment on these aspects in the text.

Thank you for this comment. I absolutely agree and we have discussed this issue in L 369 and L382. Concerning growth differences with age, increased rates are mostly visible in very young coral colonies (e.g. Carlot et al. 2021), which have not been evaluated here (average surface area 610 cm2).

A location map and an illustrative image of the study areas could be added.

A location map has now been added to the Manuscript

Explain in the discussion the importance of these studies also in the detection and evaluation of species mortality.

This possibility has been added to the discussion

Perhaps the text wording could be modified so that the sentences were in an impersonal mode?

We changed the wording in the manuscript as suggested in most of the marked sentences

One of the greatest advantages of this method is that it not only offers a growth rate per individual, but also a map that monitors spatial growth per colony is achieved.

Exactly, we are happy that this advantage came out very clear.

Discuss the importance of having biophysical parameters by species that can be related to the morphometric parameters obtained from three-dimensional models

we have added a sentence stating the importance of using species- and site-specific densities as these can vary with environmental conditions to L184

It also allows monitoring changes at the reef scale (12 -> Figuera et al., 2015)

We have added this important note as suggested.
